# Frailty in the context of COVID-19 pandemic: A life-threatening condition

**DOI:** 10.3389/fmed.2022.965562

**Published:** 2022-08-24

**Authors:** Alan L. Fernandes, Rosa M. R. Pereira

**Affiliations:** Rheumatology Division, Hospital das Clinicas HCFMUSP, Faculdade de Medicina da Universidade de São Paulo, São Paulo, Brazil

**Keywords:** frailty, SARS-CoV-2 infection, COVID-19 pandemic, older adult, mortality, health outcomes

## Abstract

The pandemic outbreak of coronavirus disease 2019 (COVID-19) has caused emerging challenges for healthcare systems regarding the assistance to the older adult population which, added to the increased life expectancy, may be exposing frail older adults to an increased risk of unfavorable health outcomes. Frailty has a pathogenesis of multifactorial etiology and is defined as a condition characterized by progressive decline in physiological function, weakness, decreased strength, and reduced resilience to stressors, leading to vulnerability and an increased risk of fractures, falls, institutionalization, and death. In the context of COVID-19, frail older adults accounted for approximately 51% of hospitalized patients with confirmed cases and elevated risk of mortality in-hospital. In addition, frailty may be associated with recent “excess mortality” reported by the World Health Organization (WHO) in terms of the full death toll associated directly (due to the disease) or indirectly (due to the pandemic's impact on health systems and society) to COVID-19. Therefore, this mini review aimed to provide a summarized discussion from meta-analyses data regarding the impact of frailty in community-dwelling older adults hospitalized with COVID-19 on short-term mortality risk.

## Introduction

The increasing life expectancy of older adults (≥ 65 years) alludes to the worldwide demographic aging in recent years, whose prospects suggest reaching 1.5 billion by 2050, almost one-sixth of the world population (1). The Global Burden of Diseases, Injuries, and Risk Factors Study (GBD) 2019, assessing 204 countries and territories between 1950 and 2019, reported a remarkable increase in healthy life expectancy (HALE) from 58.6 years in 2000 to 63.5 years in 2019 (2). Nevertheless, previous analysis of the GBD (3) revealed that a set of 92 diseases were identified as age-related, accounting for 51.3% of the entire global burden among adults in 2017.

Health status and illness severity of aging populations have been key elements to consider, in addition to chronological age (3). About 17.3% of the population aged ≥ 60 years have had functional impairments to activities of daily living (ADL), reaching 39.2% in those aged > 75 years (4). This decline may be blunting a notorious challenge to global public health, termed frailty (5), which is considered one of the most important geriatric syndromes for researchers and clinicians (6). Frailty presents a multifactorial etiology and is featured by a cumulative decline in biological reserves and functional capacities that compromise several biological systems, predisposing the individual to fractures, falls, institutionalization, and death (7).

A greater life expectancy (8) also exposes community-dwelling older adults to a high risk of frailty incidence (9), and amplify a positive and interdependent relationship between the number of cases/disease severity and an emerging need for health care services/hospitalizations (10, 11), leading to expensive costs for government agencies and greater operational demands for health services (12, 13), which have been shown to be at least two-fold as high in frail older person compared to robust ones (14).

Frailty *per se* makes older adults vulnerable to unfavorable health outcomes. In the context of a severe acute respiratory syndrome coronavirus 2 (SARS-CoV-2) pandemic, the etiological agent of coronavirus disease 2019 (COVID-19), 51.4% of patients hospitalized with positive COVID-19 were frail and had a significantly higher risk of short-term mortality compared with non-frail older adults (15). These findings corroborate previous evidence indicating frailty as an independent predictor of mortality among patients with COVID-19 (16), even in hospitalized patients younger than 65 years (17). Therefore, this study aims to provide a summarized discussion from meta-analyses data regarding the impact of frailty in community-dwelling older adults hospitalized with COVID-19 on short-term mortality risk.

## Underestimated COVID-19 deaths

An important discussion has arisen about the real number of deaths resulting from the COVID-19 pandemic. Recently, the World Health Organization (WHO) presented new estimates, termed “excess mortality,” that include full death toll associated directly (due to the disease) or indirectly (due to the pandemic's impact on health systems and society) to COVID-19, calculated at 14.9 million between January 1, 2020 and December 31, 2021 (18). Excess mortality considers the difference between the number of deaths that have occurred and the number that would be expected in the absence of the pandemic based on data from earlier years (19).

The number of deaths from COVID-19 is a discussion that precedes official government statements and depends on the model used in the analysis. On January 18, 2022, Adam (20) published in Nature a pertinent report about the efforts of countless experts to narrow the uncertainties for a global estimate of pandemic deaths through methods ranging from satellite images of cemeteries to door-to-door surveys and machine-learning computer models that try to extrapolate global estimates from available data. According to the assessment (20), global excess deaths are estimated at double or even quadruple the pandemic's official toll so far.

The Institute for Health Metrics and Evaluation (IHME) (21) suggests an increase in mortality rates of approximately 10.4 million of the 6,9 million officially reported to date, totaling a global excess COVID-19 deaths of around 17,3 million of people in the current projected scenario by August 1, 2022. This current projection is updated daily on its own modeled results, as well as projections of how quickly the global or country/territory toll might rise (21).

Indeed, deaths indirectly linked to COVID-19 have been attributable to other health conditions for which people were unable to access prevention and treatment in the overburdened health systems due to the pandemic, among which older adults were notably affected (18). It is reasonable to believe in the expressive participation of the older adult population in the aforementioned “excess mortality,” given the well-established advance of age as a natural strong risk factor (22, 23), in addition to having emerged as a key predictor of adverse outcomes in the setting of SARS-CoV-2 infection (24) since the beginning of the pandemic (25).

As of March 27, 2020, the Center for Disease Control and Prevention (CDC) (25) already reported that adults aged ≥ 65 years accounted for 31% of COVID-19 cases, 45% of hospitalizations, 53% of intense care unit (ICU) admissions, and 80% of deaths from COVID-19, with the highest percentage of severe outcomes among those aged ≥ 85 years (25). Similarly, at the same period, analogous conditions have been reported by Italy and China (26) reinforcing the pandemic impact of COVID-19 on older adults.

Little is known about the biological and environmental transition that occurs from older adults to frail ones. Frailty is not a mandatory condition as an inevitable part of aging or an irreversible one-way process to disability or death (27), but it is better understood as a dynamic and preventable process, which can also evolve positively toward reversal of the condition (27, 28).

## Highlights on frailty

Frailty deals with pathogenesis from physical to social dimensions (5), and is usually better predicted by its adverse effects than the diagnosis *per se* (29), for example, an acute illness reducing functional capacity that easily progresses to physical disability (30, 31). Frailty has been a key screening parameter to make important clinical decisions in older patients outside of the COVID-19 pandemic, such as allocating scarce health care resources (32), and successful prediction avoiding hospitalization, nursing home admission, prolonged hospital length of stay, and even death (33).

In its dynamic process, frailty status can transition between worsening and improving. The transition to a level of worse frailty is most often observed than its improvement, and the natural course of frailty development tends to be a downward spiral of increasing frailty as well as a heightened risk of worsening function, disability, falls, emergency admissions, and death (7, 28, 34). Despite affecting both sexes, frailty has been more prevalent in older adult women.

Recently, Mielke et al. (35) presented comprehensive data on the impact of gender on frailty transition. Through a 2-year prospective cohort, the authors evaluated 1,158 community-dwelling older adults with a mean age of 84.4 years. Their findings revealed that 81% (933) of participants had some degree of frailty, being 35% (401) of them frail with at least 3 out of 5 Fried's criteria (35). Frailty transition status could be determined for 1,029 of the initial 1,158 participants, with 42% (482) of them having transitioned between frailty states or death, showing improvement in 14% (160) compared to 28% (322) of worsening (35). Of those who worsened, 41% (133) died and those classified as frail at baseline were the most frequent compared to those robust ones (24 vs. 2%, respectively) (35). Furthermore, overall, in terms of worsening, men were more often than women, while in terms of improvement, women were slightly more often than men; of those who remained frail from baseline to post, women were more frequent than men (46 vs. 38%), however, frail men at baseline died more often (33 men vs. 18% women), reiterating the existence of gender differences in frailty transition rates, patterns and prediction [For details see (35)].

Aside from gender differences, frailty shows a wide variation in the existing data so far. In both developed and developing countries, the mean prevalence of frail older adults in community-dwelling residents has ranged from 11.3 to 15.7% (36–38). Data from the meta-analysis by O'Caoimh et al. (39) assessing 22 European countries reiterates these findings (12%). However, it is important to note increased variation when considering older adults in the early stage of frailty (e.g., pre-frail). In fact, the meta-analysis by Da Mata et al. (40) observed a prevalence of frailty in community-dwelling older adults ranging from 7.7 to 42.6%, while Collard et al. (22) evidenced a huge variation from 4.0 to 59.1%, very similar to those observed by Siriwardhana et al. (41) (3.9 to 51.4%) regardless of geography and economy of the countries evaluated in this study, although these values can reach 62.8% of prevalence (42).

Evidently, the broad spectrum of frailty assessment methods contributes to the variation, such as Edmonton Frail Scale (EFS), Frailty index (FI), The 5-item FRAIL scale, Study of Osteoporotic Fractures (SOF index), Cuban Frailty criteria, Brief Frailty Instrument for Tanzania (B-FIT), and physical Frailty Phenotype. The Frailty Phenotype (FP) was described by Fried et al. (7), and has been one of the most widespread and broadly accepted methods (39, 41), as well as a reproducible and feasible tool with the least variation in the prevalence of frailty (22, 41). Fried et al. (7) consider five components, of which ≥3 are defined as frail and 1 or 2 components as pre-frail, and absence as robust; they are unintentional weight loss (10 lbs. in the past year), self-reported exhaustion, weakness (grip strength), slow walking speed, and low physical activity (7).

Despite the large number of frailty assessment protocols, sarcopenia has been considered the heart of frailty and a recurrent issue among older adults. According to the European Working Group on Sarcopenia in Older People (EWGSOP2), sarcopenia is a progressive and generalized skeletal muscle disorder, manifested by a marked loss of muscle mass and strength, associated with an increased predisposition to adverse events analogous to frailty (43, 44).

Not every older adult will develop frailty, just as not every frail older adult will have sarcopenia, despite emerging evidence linking them (45). Nevertheless, it is reasonable to believe that these frail older adults may experience a set of unfavorable health outcomes pre-existing the SARS-CoV-2 infection (46) that, added to the inflammation related to COVID-19, could also aggravate earlier conditions such as reduced vitamin D (47), elevated concentrations of interleukin-1, interleukin-6, C-reactive protein (CRP), tumor necrosis factor alpha (TNF-α) and other inflammatory cytokines (48, 49), reduced blood flow in skeletal muscle, insulin resistance, functional and structural disorders in both cardiac and skeletal muscles driven by growth differentiation factor 11 (GDF11) (49, 50).

To date, there is still controversy about the impact of frailty on COVID-19, and which parameters would perform best in predicting unfavorable outcomes resulting from COVID-19, therefore, an adequate search in the available data justifies the present review.

## Frailty in COVID-19 pandemic setting

**Figure 1:**
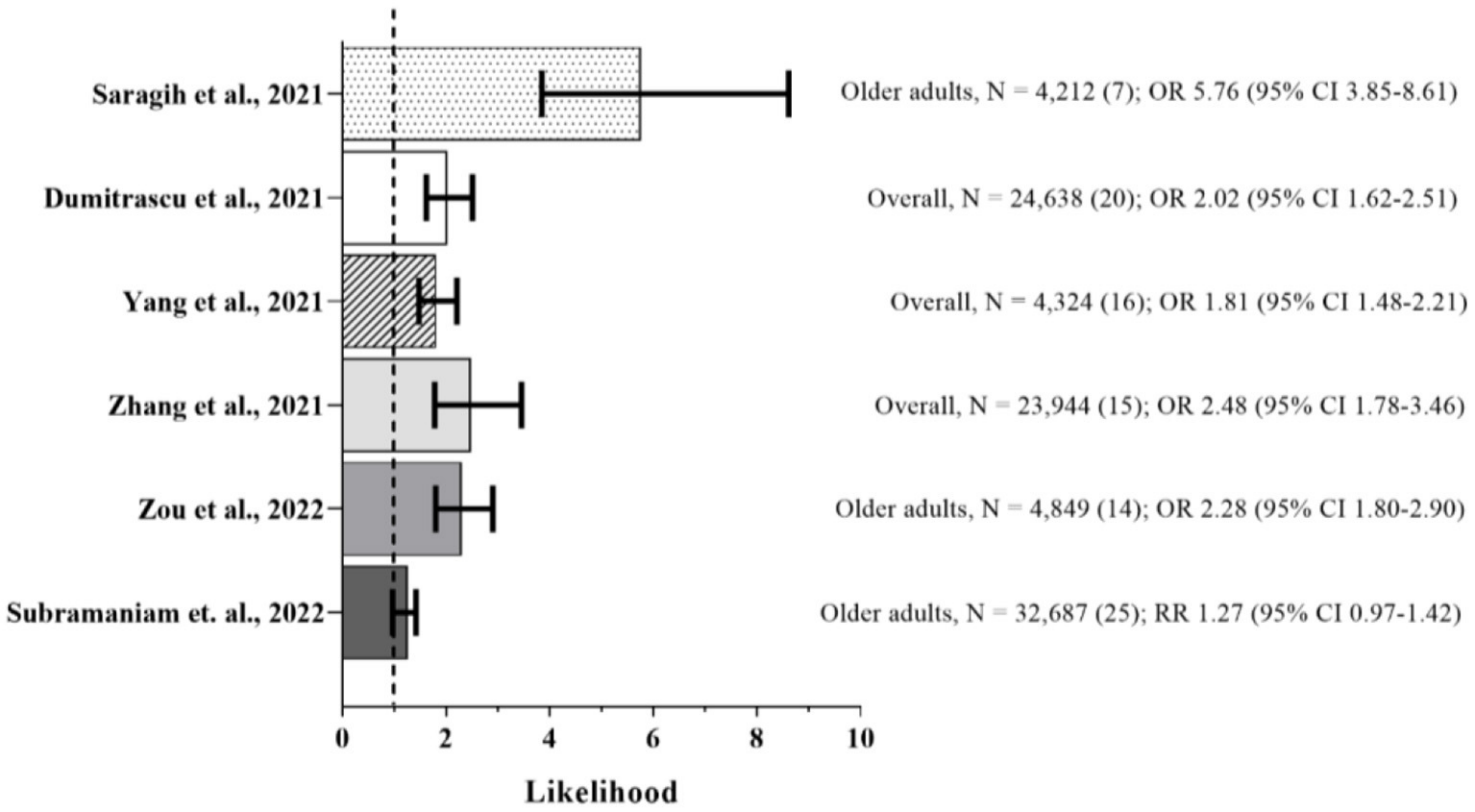
Frail vs. Non-frail Short-term Mortality. Sample, number of participants (number of studies in the meta-analysis), and likelihood of event (95% confidence interval, CI). Summary of the mortality risk outcome from meta-analyses (15, 16, 24, 51–53). Dashed line represents absence of statistical significance. Frailty was predominantly assessed by clinical frailty scale (CSF).

As part of the scope of the present review, a search was performed from PubMed database searching for meta-analyses studies reporting associations between frailty and COVID-19 related outcomes in community-dwelling older adults using the terms “frailty or frail” and “SARS-CoV-2 or COVID-19” by June 6, 2022. To date, seven studies have performed meta-analyses to assess the impact of frailty on short-term mortality risk from COVID-19 (15, 16, 24, 51–54), although Pranata et al. (54) aimed to quantify the dose-response relationship between clusters of clinical frailty scale (CFS) and mortality in COVID-19 patients. The authors (54) presented a similar distribution in the pooled prevalence for the clustered CFS 1–3 (34%), CFS 4–6 (42%), and CFS 7–9 (23%), and a significant 12% increase in mortality for each 1-point increment in CFS [OR 1.12 (95% Confidence Interval, CI, 1.04–1.20), *p* = 0.003]. Figure 1 summarizes the short-term mortality risk likelihood observed in the other studies.

Of all, the study by Saragih et al. (53) was the only one that exclusively included older adults, while Zou et al. (15) and Subramaniam et al. (51) carried out an subgroup analysis in this population. The primary outcome of all studies was short-term mortality, defined as in-hospital death or within 30 days, although three of them (24, 51, 52) also reported additional outcomes such as delirium, risk of COVID-19 severity, admission to intensive care unit, application of invasive mechanical ventilation, and long hospital length of stay.

For screening purposes for frailty in COVID-19 settings, CFS emerges as a feasible and practical tool to enable efficient workflow within hospitals, even when faced with limited human resources and increasing demand for medical services as in the pandemic era (17, 54, 55). Corroborating, a recent retrospective cohort study demonstrated that clinical frailty, assessed by CFS, was associated with late mortality in COVID-19, while features of acute infection did not show significant association (56).

When comparing CFS and Hospital Frailty Risk Score (HFRS) in predicting mortality and other adverse outcome in hospitalized patients with COVID-19, Ramos-Rincon et al. (57) showed that both tools defined frailty as an independent predictor. The HFRS measure was associated with hospital length of stay over 10 days, ICU admission and use of invasive mechanical ventilation, while CFS was associated with mortality (57), and the subgroup analysis performed by Zhang et al. (16) using the frailty assessment tool indicated that this association between death and CFS was almost two-fold higher that the other frailty tools [pooled OR 2.88 (95% CI, 1.52–5.45) vs. pooled OR 1.98, (95% CI, 1.81–2.16), respectively] such as HFRS, FI, Frail Non-Disabled Questionnaire (FNDQ), and Palliative Performance Scale.

In contrast, there is no unanimous evidence supporting an association between frailty status and mortality in patients with COVID-19 (51). Subramaniam et al. (51) observed in 21 studies composing the meta-analysis that there was an association between increased mortality risk with increasing levels of frailty, while four did not show. Despite the higher pooled mortality amongst patients with frailty compared to non-frail ones (30.6 vs. 19.4%), there was no independent increased risk of mortality [RR 1.27 (95% CI, 0.97–1.42) between frail and non-frail patients even adjusting for age and other covariates, or age stratified (51). On the other hand, based on the individual studies (*n* = 11) that composed the ICU admission outcome of this meta-analysis (51), a significant higher proportion of non-frail patients were admitted to the ICU compared with frail patients (29.1 vs. 27.2%), and those non-frail had higher mortality risk compared with frail ones [RR 1.63 (95% CI, 1.3–2.03)], as well as invasive mechanical ventilation requirement outcome.

It is important to highlight that the values foregoing in the ICU admission and mechanical ventilation outcomes are most likely justified by clinical decision-making at the highest peak of the COVID-19 pandemic, when it was necessary to choose patients with the best prognosis and greater likelihood of survival in view of scarce health resources. Patients with frailty were less commonly admitted to ICU, and those frail patients admitted were less likely to receive mechanical ventilation.

Herein, among the meta-analyses raised, the proportion of people with frailty ranged from 8.3 to 93% of the total population presented in the individual studies, while the mean pooled frailty prevalence exceeds half of the sample, ranging from 51.4 to 66% (15, 16, 24, 51, 53, 54). Overall, frail older adults with COVID-19 commonly did not survive compared to non-frail older adults, and patients who died were likely to be older and more likely to have comorbidities such as dementia, diabetes mellitus, and cardiovascular problems (24, 51, 52).

Several limitations need to be noted. First, an important share of young adults (> 18 years) may be influencing the results between survivors and non-survivors and, therefore, further studies designed to investigate those aged ≥ 65 years would be interesting. Second, there was low methodological quality and high publication bias among individual studies which, added to the researcher's subjective choice of the frailty assessment tool, contribute to the heterogeneity observed in the meta-analyses as well as to a reduction in their statistical power. Third, regardless of CFS being the most used frailty screening instrument and a reliable predictor of outcomes in acute critical care, easily performed by any trained healthcare professional, caution is needed when extrapolating the findings. CFS is a supportive diagnostic tool that complements other frailty assessment instruments and, therefore, clinical decision-making based on CFS *per se* has not been recommended (54). In addition, CFS has only been validated for older adults (≥ 65 years), it may not be suitable for younger adults (54), as noted among individual studies composing the meta-analyses.

## Conclusion

This summarized review from meta-analyses data highlights the impact of frailty in older adults hospitalized with COVID-19, showing that COVID-19 patients with frailty have an increased risk of short-term mortality compared to non-frail patients with COVID-19. The findings reinforce that frailty assessments in COVID-19 patients should be considered as an integral part of hospital screening and healthcare resource allocation, reducing short-term mortality risk, and avoiding other poor health outcomes, while aiding clinicians to manage risk-benefit approach for patients.

## Author contributions

AF drafted the manuscript. RP and AF performed the critical revision of the manuscript for important intellectual content. Both authors have read and approved the final version of the manuscript.

## Funding

This study was supported by São Paulo Research Foundation (FAPESP) (Grants Nos. 2020/11102-2, 2020/07098-0, and 2020/05752-4), Conselho Nacional de Desenvolvimento Científico e Tecnológico (CNPq) (Grant No. 305556/2017-7), and Coordenação de Aperfeiçoamento de Pessoal de Nível Superior–CAPES (Grant No. 88887.507119/2020-00).

## Conflict of interest

The authors declare that the research was conducted in the absence of any commercial or financial relationships that could be construed as a potential conflict of interest.

## Publisher's note

All claims expressed in this article are solely those of the authors and do not necessarily represent those of their affiliated organizations, or those of the publisher, the editors and the reviewers. Any product that may be evaluated in this article, or claim that may be made by its manufacturer, is not guaranteed or endorsed by the publisher.
